# Imagined eye cue increased altruistic behavior toward charity instead of stranger

**DOI:** 10.3389/fpsyg.2025.1503766

**Published:** 2025-03-11

**Authors:** Jieyu Lv, Yuanya Zhang, Yuxin Shen, Xuedong Weng, Liang Xu

**Affiliations:** ^1^Department of Psychology, School of Sociology and Psychology, Central University of Finance and Economics, Beijing, China; ^2^Mental Health Education Center, Lishui Vocational and Technical College, Lishui, Zhejiang, China

**Keywords:** watching eyes effect, imagined eye cue, altruistic behavior, a sense of being seen, dictator game

## Abstract

Previous research has not established a significant link between imagined eye cue and altruistic behavior, nor has it verified whether a sense of being seen played a role in it. This study employed a between-subjects design with a single factor (Cue Type: Imagined Eye Cue/Imagined Flower Cue/No Cue) to explore the impact of imagined eye cue on individuals' altruistic behavior in two different dictator games, and also assessed the mediating role of a sense of being seen. It revealed that participants who was presented with imagined eye cue acted more altruistically than those who was presented with imagined flower cue or no cue when the recipient of the dictator game was a charity. Although imagined eye cue strengthened participants' a sense of being seen, this sense did not mediate the relationship between cue type and altruistic behavior. The findings suggest that the imagined eye cue may encourage individuals to donate generously by stimulating their internal social norms. This provides a theoretical rationale for the normative mechanisms underlying the watching eyes effect and explores a more cost-effective and accessible approach for interventions aimed at promoting charitable behavior.

## 1 Introduction

In an increasingly interconnected world, people are confronting significant challenges, including the rapid spread of deadly diseases and large-scale refugee crises. Altruism plays a crucial role in fostering a sense of global community and shared responsibility (Yang et al., 2020). Individuals can contribute to addressing these issues by donating resources to provide medical aid or assisting refugees in their integration into new communities (Klimecki et al., 2016). Therefore, encouraging and promoting altruistic behavior among individuals contributes to the creation of a more harmonious and sustainable future.

Watching eyes effect refers to the phenomenon in which individuals undergo behavioral changes when they are looked at by another person or when they see eye-like figures (Nettle et al., 2013). Research has demonstrated that cues related to eyes can effectively enhance altruistic behavior (Lv et al., 2025). For instance, a study conducted a pivotal experiment using a dictator game that revealed participants who were exposed to images of eyes exhibited greater generosity than those who viewed images of flowers (Haley and Fessler, 2005). This phenomenon has been consistently supported by subsequent studies, which have shown that various eye representations–ranging from real human presences (Izuma et al., 2011) to stylized images (Wang and Dai, 2020)–significantly elevate altruistic behavior. Field experiments have similarly indicated that eye cues can positively influence actions such as paying through honesty boxes (Bateson et al., 2006), reducing theft (Nettle et al., 2012), decreasing littering (Bateson et al., 2013), encouraging voting (Panagopoulos, 2014), increasing blood donation (Sénémeaud et al., 2017), and minimizing fare evasion (Ayal et al., 2021). Moreover, the watching eyes effect has practical applications in various fields, including architectural design (Dear, 2018). Collectively, these findings underscore the significant impact of the watching eyes effect on altruistic behavior across diverse settings.

Although the watching eyes effect has been documented in numerous laboratory and field experiments, its replicability remains a subject of debate (Tane and Takezawa, 2011; Sparks and Barclay, 2015; Rotella et al., 2021). For instance, a meta-analysis failed to demonstrate a significant impact of artificial monitoring cues on generosity as measured by the dictator game or public goods game (Northover et al., 2017). However, in another study, the watching eyes effect was observed solely in the dictator game among four economic game paradigms (Lv et al., 2025). These findings suggest that the stability of the watching eyes effect is questionable. A recent meta-analysis (Wang et al., 2023) also highlighted that the role of the watching eyes effect varies across different contexts and types of behavior. This inconsistency may arise from the fact that the manifestation of the watching eyes effect is constrained by multiple factors. Research indicates that the effectiveness of the watching eyes effect depends on specific contextual elements (Fehr and Schneider, 2010; Cai et al., 2015) and individual differences (Vogt et al., 2015; Rotella et al., 2021). Therefore, while eye cues appear to promote altruistic behavior to some extent, the universality of their effect still requires further investigation.

Traditionally, studies have focused on basic cognitive processes, such as auditory and visual presentations of eye-related cues. However, a recent study utilized higher-order cognitive processes to manipulate eye cues (Lv et al., 2024). Their findings indicated that participants in a dictator game exhibited more altruistic behavior in imagined scenarios than when exposed to visual cues. Notably, however, the study found no significant difference in altruistic behavior between scenarios involving imagined eye and those involving imagined flower, implying that imagined eye cue may not be a determinant of altered altruistic behavior. Instead, it appears that imagery alone can enhance altruism. To further investigate the role of imagined eyes and their psychological mechanisms while controlling for the potential influence of flower imagery materials, the present study builds upon prior research by incorporating a no cue to establish a baseline. This addition aims to elucidate whether the effect of imagined eye cue on altruistic behavior is enhanced or diminished. Accordingly, we propose Research Hypothesis 1: *Altruistic behavior will be higher in the imagined eye cue compared to both the no cue and the imagined flower cue, imagined eye can promote individual's altruistic behavior*.

The traditional dictator game (TDG), as a classic tool for exploring altruistic behavior, focuses on individuals' autonomous decision-making in resource allocation. While this paradigm has methodological advantages, its laboratory nature may undermine its real-world explanatory power. First, in real-world contexts, the majority of individual donations are made through charitable organizations rather than directly to individuals (Charities Aid Foundation, 2022). Charitable organizations often act as intermediaries to coordinate the distribution of resources to ultimately benefit the end recipients (Bekkers and Wiepking, 2011). The binary decision structure in the TDG (allocator-recipient) struggles to fully replicate this real-world decision-making mechanism. Second, the fixed-amount allocation task cannot capture the dynamic trade-offs in real philanthropic decision-making. As emphasized by Livingston and Rasulmukhamedov (2023), who notes the risk of diminished explanatory power when predicting real-world social behavior using the traditional paradigm. To overcome this limitation, some researchers have enhanced the paradigm's reality correspondence through innovation (Izuma et al., 2011). The core breakthrough of this innovative design lies in expanding the resource allocation targets to real charitable organizations and introducing flexible donation proportion choices (rather than fixed-amount allocations). This more accurately simulates the multidimensional considerations in real donation decisions–participants must weigh their personal retained earnings while simultaneously assessing the actual support received by philanthropic causes. To establish a more comprehensive validation framework, this study employs a dual-dimension experimental design: retaining the methodological strengths of the traditional paradigm while incorporating this innovative one. The complementary design of the paradigms to systematically test the situational universality of the watching eyes effect is an innovation of this study. Although the replicability of the watching eyes effect in the TDG remains academically controversial (Sparks and Barclay, 2015; Northover et al., 2017), there is some empirical support within this paradigm (Haley and Fessler, 2005; Rogers et al., 2014; Lv et al., 2025). Meanwhile, in the more ecologically valid innovative dictator game (IDG), such as the study by Izuma et al. (2011) that incorporates a charitable donation mechanism into the decision-making framework, the presence of an observer significantly increases donation levels. Based on these dual evidence bases, we propose Research Hypothesis 2: In the TDG, the imagined eyes cue can promote altruistic behavior; in the IDG, the imagined eyes cue can also promote altruistic behavior.

According to Indirect Reciprocity Theory, eyes serve as social presence cues that convey information about the attention and evaluation of others, which motivates individuals to enhance their status in social groups by displaying positive images (Sylwester and Roberts, 2013). That is, people will tend to convey positive signals to enhance their reputation by displaying altruistic behaviors when they know they may be observed by others. This is the well-known reputation mechanism in the field of the watching eyes effect, which emphasizes an individual's sensitivity to potential observational cues. Empirical evidence supports the notion that an awareness of being seen influences behavior; individuals are motivated to uphold their reputations when prompted by eye cues (Burnham and Hare, 2007; Oda et al., 2011). Further research has pointed out that eye cues are effective in inducing an individual's a sense of being seen (Pfattheicher and Keller, 2015), and the observability of behavior has a positive effect on pro-social behavior (Bradley et al., 2018). Specifically, individuals who perceive themselves as being observed are more likely to engage in pro-social behaviors in order to maintain their social reputation. However, many existing studies have employed simplistic comparative methods that only assess differences between observed and unobserved states, failing to explore how a sense of being seen quantitatively influences altruism. For example, many studies have focused on exploring the association between eye cues and behavioral changes, such as an increase in generosity or a decrease in stealing behavior, but few studies have directly examined whether eye cues actually enhance an individual's a sense of being seen (Dear, 2018). This limitation may overlook the complex psychological dynamics underlying social behavior. Recognizing this gap, the present study incorporates established quantitative measures of a sense of being seen (Pfattheicher and Keller, 2015) and introduces a straightforward self-report tool to investigate whether imagined eye cue can instigate behavioral changes by evoking an individual's a sense of being seen. An innovative aspect of this research is the treatment of “a sense of being seen” as an independent variable, aiming to create a more comprehensive framework for understanding the psychological mechanisms involved. Accordingly, we propose Research Hypothesis 3: *A sense of being seen mediates the relationship between imagined eye cue and individual altruistic behaviors*.

## 2 Method

### 2.1 Participants

The sample size was determined using G*Power 3.1 software (Faul et al., 2007), employing an *F*-test based on a one-way three-level between-groups ANOVA. This approach was consistent with a *post-hoc* test effect size *f* = 0.365 as established in prior research (Lv et al., 2024), with a significance level α = 0.05. Consequently, a total of 78 groups (156 participants: Each group included one recipient and one dictator) were required to achieve a statistical power of 1−β = 0.80. A total of 168 participants were recruited for this study. However, six participants did not answer the test questions correctly, resulting in a final count of 162 valid participants (*M*_age_ = 21.98, *SD*_age_ = 3.31; 98 females and 64 males). The study received approval from the Research Committee of the School of Sociology and Psychology at the Central University of Finance and Economics, and informed consent was obtained from all participants. Each participant was compensated with a fixed payment of 10 yuan (equivalent to $ 1.41), plus an additional payment ranging from 0 yuan to 5 yuan, contingent upon the decisions they made during the experiment.

### 2.2 Design

A one-factor (Cue Type: Imagined Eye Cue/Imagined Flower Cue/No Cue) between-subjects experimental design was employed in this study. The dependent variable was the amount of tokens allocated to the recipient by the dictator in the dictator game. Control variables included demographic factors such as gender and age. Two paradigms of the dictator game were utilized: the TDG and the IDG. To ensure the reliability and validity of the measurements, the study implemented two approaches to assess participants' a sense of being seen. The first approach followed previous research based on the spotlight effect (Pfattheicher and Keller, 2015), while the second approach utilized a self-report method. This dual assessment strategy aims to provide a comprehensive understanding of how the imagined eye cue influences altruistic behavior in the context of the dictator game.

### 2.3 Materials

#### 2.3.1 Traditional dictator game

In the TDG, conducted over a single round and anonymously, participants are assigned to one of two roles: dictator or recipient. The roles are randomly allocated by the system. The dictator has the authority to decide how many of the 100 tokens to assign to the recipient, while the recipient does not have the option to refuse the allocation; they can only accept the proposed distribution. All remaining tokens are retained by the dictator.

#### 2.3.2 Innovative dictator game

**Figure 1:**
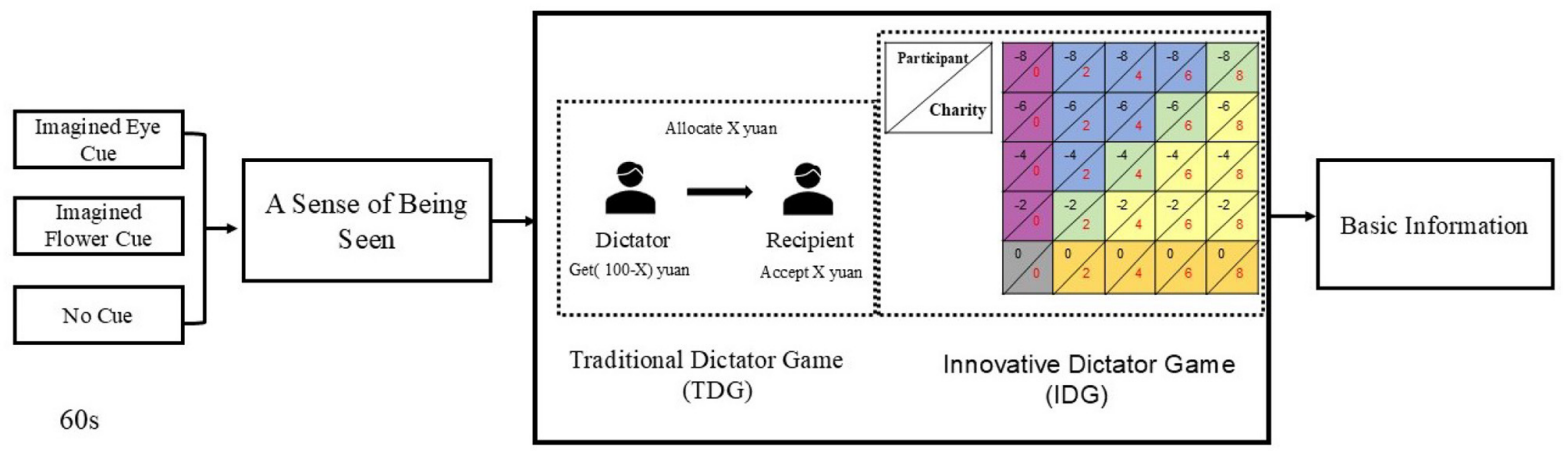
Experimental procedure.

In contrast to the TDG, the IDG alters the roles such that all participants assume the role of recipient. Participants are informed that they will receive a specific amount of money and will then face a series of choices regarding whether to donate some or all of this amount to a charity (Izuma et al., 2011) or to an individual (Cage et al., 2013). In this study, each participant was “allocated” 8 RMB (equivalent to $1.13) and was informed of the opportunity to use this money to support charitable donations. They were subsequently presented with the decision of whether to forgo a portion of their funds to make a donation. The payoff matrix reflecting participants' potential losses and rewards is depicted in Figure 1. In each cell of the matrix, the number in the upper-left corner indicates the amount of money lost by the subject, while the number in the lower-right corner represents the amount of money received by the charity. In the IDG, choosing “I do"indicates that the subject engages in altruistic behavior, defined as “1;” choosing “I refuse” indicates that the subject engages in egoistic behavior, defined as “0.” The 0-0 decision was excluded from the analysis (Figure 1, subject loss 0 yuan, and the charity received 0 yuan), the 24 choices were summed up, and the larger the number, the more altruistic the participant was.

#### 2.3.3 Measurement of a sense of being seen

The first measure draws on previous research (Pfattheicher and Keller, 2015), in which participants were hypothetically asked to envision walking through a hall filled with 30 individuals while wearing a T-shirt featuring a pop star. They were then asked to estimate how many people would notice their T-shirt. The participants' a sense of being seen was quantified by the number of individuals they believed would take notice, with findings indicating that eye cues significantly enhanced this sense. The present study employed a similar design to investigate whether imagined eye cue could elicit comparable effects, which we refer to as the “Spotlight Effect Method” for convenience. Furthermore, considering the cultural differences between Eastern and Western societies, measures applicable to individualistic cultures may not accurately reflect the true feelings of participants from collectivistic cultures. Thus, alongside the Spotlight Effect Method, the present study also utilized a Self-Report Method to gauge participants' a sense of being seen, aiming to more precisely capture this subjective experience. The Self-Report Method was implemented using a 7-point Likert scale, prompting participants to indicate the extent to which they “*feel that someone is watching me*.”

### 2.4 Procedure

Participants were recruited via an online format and completed the experiment remotely on the oTree platform. To participate, participants were required to log in to a predetermined website, such as http://49.233.63.61//room/room1. Each experimental room had a unique link (e.g., the URL for room 5 ends with “room5”), and each room accommodated two participants, who were randomly paired. Participants first reviewed the study information and signed an informed consent form, subsequently waiting for another participant to complete the form before commencing the experiment. The entire experiment was conducted in four steps, as illustrated in Figure 1.

In the preliminary phase, participants were instructed to imagine specific cues for one minute: “*Please imagine that a pair of eyes is watching you!*” (Imagined Eye Cue), “*Please visualize a flower in full bloom before you!*” (Imagined Flower Cue), or “*Please envision completely emptying your mind!*” (No Cue). The presentation time lasted 60 seconds.

The assessment of a sense of being seen. The first assessment referred to as the Self-Report Method asked participants to rate their agreement with the statement, “*I feel that someone is watching me*” on a scale from 1 (*not at all consistent*) to 7 (*completely consistent*). The second assessment, the Spotlight Effect Method, required participants to imagine a specific scenario: “*As part of the ‘Attention' study, you are asked to wear a T-shirt featuring a picture of Andy Lau (a well-known movie star). This study takes place in another room at the school, and to reach it, you must pass through a hall where 30 people are present. How many of these 30 individuals do you think will notice your T-shirt? Please enter your estimate (0–30) in the text box below*.”

After completing the assessments, participants proceeded to the TDG, where roles (dictator or recipient) were assigned randomly by the system. The dictator and recipient were able to communicate through an online dialogue box, and after this communication, the dictator decided how many of the 100 tokens to retain for themselves.

Then, participants engaged in the IDG, taking on the role of recipients. They were told they would receive 8 RMB and were presented with the option to donate a portion of this amount to a farming program, with the amount received by the farming program varying. Each participant tested each cell of the benefit matrix and had the choice of either “*I do*” or “*I refuse*.” The 25 trials were randomized.

At the final stage of the experimental procedure, participants were required to submit their personal information. Upon completion of the experiment, each participant will receive 10 RMB (equivalent to $1.41) as a participation fee, in addition to an additional reward of between 0 and 5 RMB depending on the decisions they make in the IDG.

## 3 Results

### 3.1 Altruistic behavior of dictators in TDG

**Figure 2:**
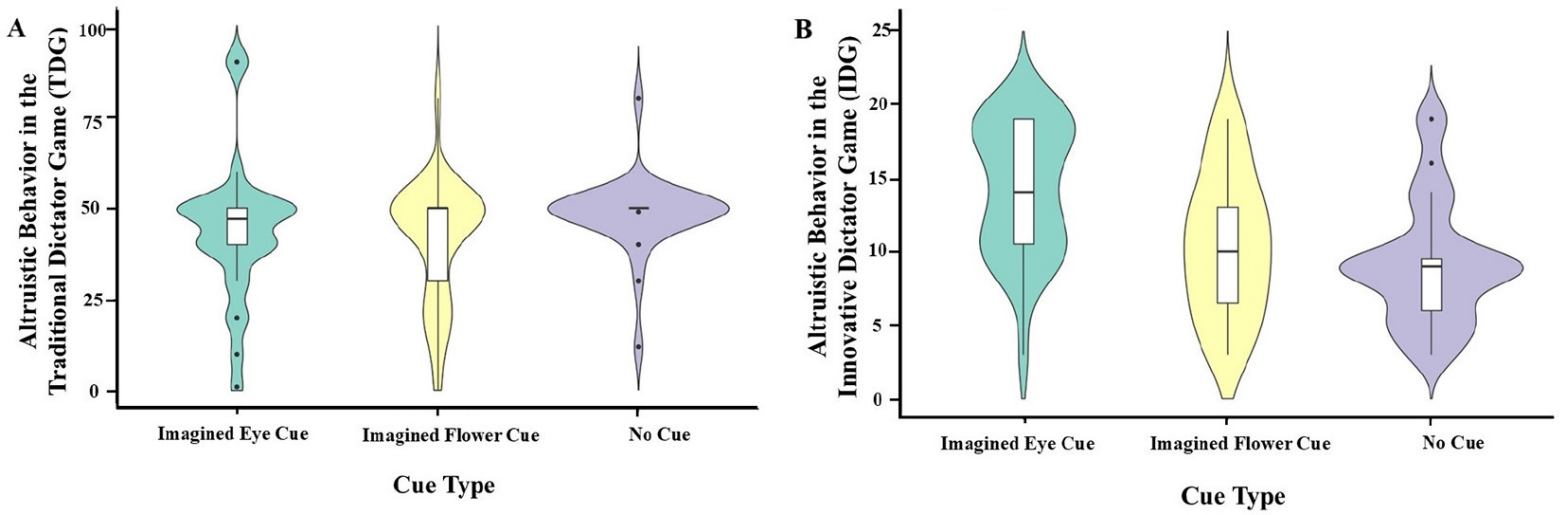
The effect of imagined cues on dictator's altruistic behavior in two dictator games. **(A)** Altruistic Behavior in the Traditional Dictator Game (TDG); **(B)** altruistic Behavior in the Innovative Dictator Game (IDG).

Table 1 presents the results of descriptive statistics for the values assigned by the dictators in the three experimental conditions of the TDG.^1^ The results indicate that the mean allocation value in the no cue of the TDG is greater than that of the imagined eye cue, which in turn is higher than that of the imagined flower cue. The variables were subjected to the Shapiro-wilk normality test revealed that the assigned values did not conform to normal distribution (*W* = 0.79, *p* < 0.001) while the results of the variance chi-square test showed that the data were not homogeneous in terms of variance [*F*_(2, 79)_ = 3.48, *p* = 0.04], which necessitated the use of nonparametric tests. The results of the Kruskal-wallis rank sum test showed that there was no significant effect of different cue types on dictator altruistic behavior under the imagined conditions [χ^2^ = 4.21, *df* = 2, *p* = 0.12]. Analysis of the two-by-two groups using the Pairwise-wilcox nonparametric test (Figure 2), with the bonferroni method chosen for *p*-value correction, showed no significant differences in dictator altruistic behavior between the imagined eye cue and the no cue (*p* = 0.15), the imagined flower cue and the no cue (*p* = 0.30), and the imagined eye cue and the imagined flower cue (*p* = 1.00).

**Table 1:** Allocation and a sense of being seen of dictator under three experimental conditions.

**Experimental condition**	**Altruistic behavior**					**A sense of being seen**			
	**Traditional dictator Game**			**Innovative dictator game**		**Self-report method**		**Spotlight effect method**	
	*n*	*M*	*SD*	*M*	*SD*	*M*	*SD*	*M*	*SD*
Imagined eye cue	27	42.89	7.78	14.22	4.5	5.07	1.27	16.63	7.78
Imagined flower cue	27	42.12	7.84	10.37	4.76	4.07	1.94	19.74	7.84
No cue	28	48.19	7.59	9.18	4.24	3.43	1.62	15.07	7.59

### 3.2 Altruistic behavior of dictators in IDG

The descriptive statistics of the dictator's decisions in the three experimental conditions of the IDG are shown in Table 1. A Shapiro-wilk normality test on the variables revealed that the values did not conform to a normal distribution (*W* = 0.94, *p* < 0.001), and the results of the test of homogeneity of variances showed that the data were variance homoscedastic [*F*_(2, 79)_ = 1.62, *p* = 0.20]. Using the nonparametric test Kruskal-wallis rank sum test, the results showed a significant effect of different cue types on participant altruistic behavior under imagined conditions (χ^2^ = 17.05, *df* = 2, *p* < 0.001). This result diverges from the findings obtained in TDG.

Analysis of the two groups using the Pairwise-wilcox nonparametric test (e.g., Figure 2), the results showed that the altruistic behavior of the participants in the imagined eye cue was significantly higher than that of the no cue (*p* < 0.001) and also significantly higher than that of the imagined flower cue (*p* = 0.013), whereas there was no significant difference between the imagined flower cue and the no cue (*p* = 0.92).

### 3.3 The mediating role of a sense of being seen

Table 1 presents the measured data of participants' a sense of being seen under three different experimental conditions. A Shapiro-wilk normality test was performed on the observed sensations measured in the two different ways and found that they did not conform to a normal distribution (Self-Report Method: *W* = 0.91, *p* < 0.001; Spotlight Effect Method: *W* = 0.95, *p* = 0.005), and the results of the homogeneity of variances test showed that the variance of the observed sensations data is not homogeneous in the Self-Report Method [*F*_(2, 79)_ = 4.33, *p* = 0.02], and the variance of a sense of being seen data under the Spotlight Effect Method of measurement is homogeneous [*F*_(2, 79)_ = 0.03, *p* = 0.97]. Results showed that participants' a sense of being seen was significantly different across cue types when measured by the Self-Report Method using the non-parametric test Kruskal-wallis rank sum test (χ^2^ = 11.5, *df* = 2, *p* < 0.001). The cues were analyzed using the Pairwise-wilcox non-parametric test was performed to analyze the two-by-two groups, and the results showed that the imagined eye cue (*M* = 5.07, *SD* = 1.27) vs. the no cue (*M* = 3.43, *SD* = 1.62) were observed to be significantly different(*p* < 0.001), the between the imagined flower cue (*M* = 4.07, *SD* = 1.94) and the imagined eye cue (*p* = 0.24), and between the imagined flower cue and the no cue (*p* = 0.67) were not significantly different from each other. A sense of being seen for participants under different cue types was not significant when measured by the Spotlight Effect Method using the nonparametric test Kruskal-wallis rank sum test (χ^2^ = 5.30, *df* = 2, *p* = 0.07). Analysis of the two-by-two groups using the Pairwise-wilcox nonparametric test. The results showed no significant differences between the imagined flower cue (*M* = 19.74, *SD* = 7.84) and the no cue (*M* = 15.07, *SD* = 7.59), *p* = 0.08. There is no significant differences between the imagined eye cue (*M* = 16.63, *SD* = 7.78) and the imagined flower cue (*p* = 0.42), and between the imagined eye cue and the no cue (*p* = 1.00).

Using dictator allocation in the IDG as the dependent variable (Y), imagery cue type as the independent variable (X), and a sense of being seen as the mediator variable (M), with the addition of the control variables of gender and age, relative mediation analyzes were utilized due to the fact that the independent variables were multicategorical categorical variables (Hayes and Preacher, 2014). In conjunction with R-mediation, 1,000 samples were sampled by Bootstrap method to estimate the 95% confidence intervals were used to test for mediating effects (Tingley et al., 2014). According to the recommendation of the study (Fang et al., 2017), 1−α/(*k*−1) was used instead of the usual 1−α in order to reduce the Type I error. Therefore, the significance level of the relative mediation effect in this study was 0.017.

**Figure 3:**
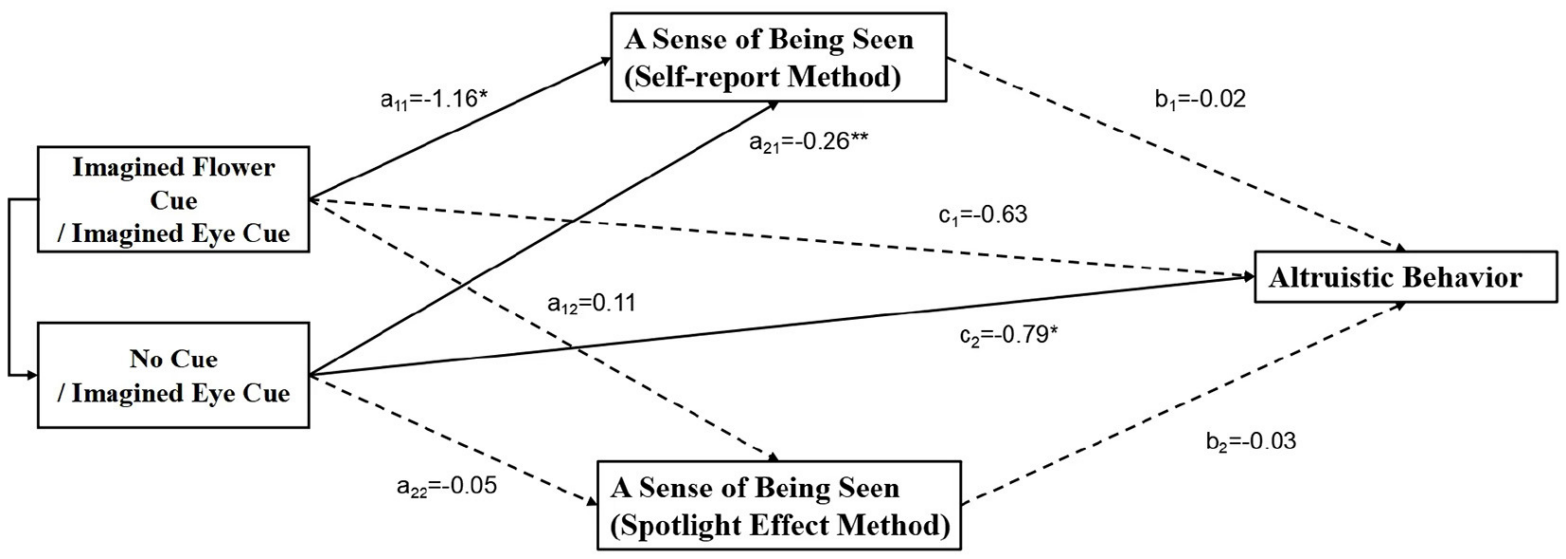
Mediator model coefficients for a sense of being seen for both measurements. *Indicates *p* < 0.05 and **indicates *p* < 0.01.

The results of the hierarchical regression analysis of the variables are shown in Figure 3, participants in the imagine flower cue and the no cue were less likely to feel observed as measured by the Self-Report Method compared to the imagine eye cue, [*a*_11_ = −0.16, *p* = 0.04, *t*_(79)_ = −2.13;*a*_21_ = −0.26, *p* = 0.001, *t*_(79)_ = −3.40]. Although the imagine flower cue perceived more observations and the no cue perceived fewer observations under the Spotlight Effect Method, there was no statistical significance [*a*_12_ = 0.11, *p* = 0.17, *t*_(79)_ = 1.38;*a*_22_ = −0.05, *p* = 0.53, *t*_(79)_ = −0.63]. Therefore, a further test of relative mediation effects can be conducted on the observed sense of Self-Report Method. The results showed that the 95% confidence intervals for the relative mediated effects of the imagine flower cue and the no cue, using the imagine eye cue as a reference, included 0, and the relative mediated effects were not significant. The effect sizes for the relative direct effect and relative mediated effect are shown in Table 2.

**Table 2:** The mediating effect of a sense of being seen measured by Self-Report Method.

**Conditions**	**β**	***SE***	***p***	**95%CI**	**η**
**Imagined flower cue-imagined eye cue**					
Relative direct effect	–0.234*	0.08	0.004	[-0.39,–0.07]	-
Relative mediation effect	0.001	0.04	0.99	[–0.06,0.06]	–0.43%
Relative total effect	–0.233*	0.08	0.004	[–0.39,–0.07]	-
**No cue-imagined eye cue**					
Relative direct effect	–0.294***	0.04	< 0.001	[-0.45,–0.12]	-
Relative mediation effect	0.001	0.06	0.99	[–0.08,0.07]	0.34%
Relative total effect	–0.293**	0.09	0.002	[–0.43,–0.14]	-

*Indicates *p* < 0.05, **indicates *p* < 0.01, and ***indicates *p* < 0.001.

## 4 Discussion

This study investigated the influence of imagined eye cue on altruistic behavior through a controlled laboratory experiment, focusing on behavioral differences across two dictator game paradigms and examining the mediating role of a sense of being seen. Results demonstrated that participants in the imagined eye cue exhibited significantly higher donation intentions toward charities in the IDG compared to the imagined flower cue and no cue, whereas no significant differences emerged in the TDG. Although imagined eye cue enhanced self-reported sense of being seen, this sense did not mediate altruistic behavior changes. The findings suggest that the effect of imagined eyes may stem from the activation of internalized social norms rather than reputation management. These results provide novel evidence for normative explanations of the watching eyes effect and highlight the potential utility of imagined cues in real-world charitable contexts.

### 4.1 Effect of imagined eye cue on the participants' altruistic behavior

In the IDG, the altruistic behavior exhibited by participants in the imagined eye cue was significantly greater than that of both the imagined flower cue and the no cue. This finding substantiates Research Hypothesis 1, indicating that imagined eye cue can elicit the watching eyes effect similar to that produced by actual eye cues, thereby promoting an individual's altruistic behavior. Furthermore, there was no significant difference in the altruistic behavior of participants in the imagined flower cue compared to the no cue. This suggests that the increase in participants' altruistic behavior was attributable to the imagined eye cue rather than the imagery itself. However, in the TDG, the altruistic behavior of participants under the imagined eye cue was not significantly different compared to the no cue. This part of the results does not support Research Hypothesis 1.

### 4.2 Variations in participants' altruistic behavior in response to different recipients

In the TDG, the differences in altruistic behaviors among dictators across experimental conditions were not statistically significant. However, in the IDG, participants in the imagined eye cue demonstrated significantly higher levels of altruistic behavior compared to those in the other two groups, thereby partially confirms Research Hypothesis 2. Contrary to our initial hypothesis, participants in the TDG did not exhibit more altruistic behavior under the imagined eye cue.

Previous research has shown that donation amounts increase markedly-up to double-when participants know that recipients in the dictator game are charities instead of anonymous individuals (Eckel and Grossman, 1996). The disparity in results between the two dictator game paradigms may stem from their differing designs. In the IDG, recipients were identifiable charities that organized aid events, contrasting with the anonymous nature of recipients in the traditional setup. This shift in design likely influenced participants' motivations and decision-making processes, contributing to the observed inconsistencies in altruistic behavior across the two formats.

The differences in outcomes across these two dictator game paradigms can be explained through reputational and normative mechanisms. From a reputational perspective, individuals may believe that donations to charities will enhance their social standing (Cage et al., 2013). This belief is anchored in the concept of indirect reciprocity (Nowak and Sigmund, 2005), where individuals who have previously helped others are likely to receive assistance in return. To ensure that indirect reciprocity continues to promote altruistic behavior, an individual's helping behavior needs to be able to be observed or indirectly known through social communication mechanisms (e.g., small talk) (Sommerfeld et al., 2007), which underscores the importance of behavioral visibility for reputation construction (Bradley et al., 2018). Charitable contributions often involve a public commitment, signaling social responsibility and generosity. This visibility allows for greater acknowledgment and validation of one's contributions, resulting in positive reputational feedback (Karlan and McConnell, 2014). When individuals imagine being observed by eyes, their attention to social evaluations may increase, making charitable giving more appealing. Hence, differences in donation behaviors toward charities were more pronounced in the imagined cue conditions compared to behaviors directed at anonymous strangers.

From the perspective of social norms, donating to charities is a recognized altruistic behavior aligned with societal expectations. By prompting participants to imagine the presence of eyes, researchers create a simulated context of social scrutiny, potentially activating internal social norms related to altruism and responsibility (Zhang and Liu, 2017). When participating in the dictator game with a charity as the recipient, individuals may experience a diminished perceived normative illusion (Shi et al., 2022). They often believe that their altruistic behavior is not only accepted but also widely supported and encouraged. This perceived normative illusion can be amplified through imaginative manipulation, leading participants to assume that their actions will be positively evaluated by society, thus increasing their altruistic tendencies. Consequently, imagined eye cue can motivate individuals to align their decisions with social expectations by reinforcing their perceptions of social norms. In real-life charitable scenarios, individuals may contemplate whether their actions will be praised by society. If they perceive that charitable giving is encouraged and supported, they are more likely to engage in such behavior.

Additionally, the Loss-Reward Incentive Model also provides a powerful analytical framework. When individuals are faced with an altruistic behavioral decision, they naturally evaluate potential losses against possible rewards (Zhang and Liu, 2017). In other words, individuals in altruistic behavioral situations are not only passively influenced by normative and reputational mechanisms, but also actively consider the likelihood of social feedback and the consequences of their behavior and make decisions accordingly. For instance, while a one-time altruistic act toward a stranger may be viewed as a goodwill gesture, it carries the risk of not being recognized or appreciated, thereby limiting the expected social rewards. Conversely, donations to charitable organizations significantly reduce this uncertainty. By choosing to donate, individuals can ensure that their contributions serve the public good, making this form of altruism more appealing. Under the influence of imagined eye cue, individuals may be more inclined to donate to charities, as this behavior is likely to yield positive social feedback and enhance their personal reputation.

In addition to the explanations mentioned above, there are also explanations based on participants'emotions that can account for the different results in the two paradigms. For instance, images of eyes can elicit negative emotions (Panagopoulos and van der Linden, 2017), which in turn may lead individuals to engage in more prosocial behaviors to alleviate such emotions (Schacter and Margolin, 2019; Aknin et al., 2018). In the TDG, where the recipient of the donation is a stranger and anonymous, participants may experience relatively lower levels of negative emotions (such as anxiety and guilt). This is because anonymity reduces participants'empathy and sense of responsibility toward the recipient. However, in the IDG, where the recipient is a charitable organization, participants may more readily perceive the positive social impact of their actions, thereby generating a stronger drive of negative emotions (such as guilt and anxiety), prompting them to donate to mitigate these feelings. It should be noted that in this study, we did not measure participants' emotions. Future research could investigate whether imagined eyes cues elicit negative emotions in participants.

In everyday life, individuals frequently confront decisions about supporting charitable initiatives, making IDG particularly valuable for simulating these contexts. By analyzing dictators' decisions in such scenarios, researchers can better understand the psychological mechanisms that drive altruistic choices in real-world situations.

### 4.3 The mediating role of a sense of being seen

The results of the experiment did not support Research Hypothesis 3. The study did not replicate findings from prior research (Pfattheicher and Keller, 2015), as the differences in participants' a sense of being seen across groups were not significant under the spotlight effect measurement approach. This may be attributed to cultural contexts, where individuals in collectivist societies might be more attuned to the perceptions and evaluations of others. In this case, the imagined task associated with the spotlight effect could have elicited a strong sense of being seen, thus rendering subtle eye cues ineffective in enhancing this feeling. Consequently, no significant differences were observed across experimental groups. Nevertheless, a sense of being seen of the imagined eye cue was significantly higher than that of the no cue under the Self-Report Method, proving that the imagined eye cue were effective in eliciting a sense of being seen of the participants. However, further statistical analyses showed that the mediating role of a sense of being seen between imagined eye cue and altruistic behavior was not significant.

In other words, the watching eyes effect elicited by imagined eye cue may be more reliant on normative mechanisms than on reputational mechanisms. The Culture-gene co-evolution theory posits that throughout the evolution of human societies, individuals have developed psychological mechanisms that facilitate adherence to social norms (Chudek and Henrich, 2011). People are inclined to act in accordance with the rules present in their environment (Kawamura and Kusumi, 2017) and to impose sanctions on those who violate these rules (Fehr and Schneider, 2010). Research in the domain of substantive eye cues has provided evidence supporting the notion that the watching eyes effect promotes pro-social behavioral adherence through normative mechanisms. For instance, the combination of eye images with a written appeal stating “please don't litter” proved to be more effective in reducing littering behavior in a university cafeteria than when eye images were paired with irrelevant messages (Ernest-Jones et al., 2011). Furthermore, the placement of eye images on bike racks, accompanied by a moral reminder (“*We're watching you, bike thief!*”), has also been shown to effectively deter theft (Nettle et al., 2012).

Normative psychology posits that normative mechanisms serve as psychological and behavioral regulation processes grounded in individuals' internalization of social rules and expectations. From this perspective, while both reputational and normative mechanisms provide robust explanations for the watching eyes effect, imagined eyes do not constitute a threat to an individual's reputation at the cognitive level of imagination. Instead, it is the social norms that have been deeply internalized within individuals that truly influence their altruistic behavior. This elucidates why imagined eye cue can evoke a sense of being seen in participants, yet the alteration in their altruistic behavior is not directly instigated by this sense.

### 4.4 Limitations and prospects

This study has several limitations. Firstly, while our study attributed the divergent results between the TDG and IDG to recipient identity (anonymous stranger or charity), it is critical to acknowledge that multiple design differences between the two paradigms may jointly influence the manifestation of the watching eyes effect. For instance, in terms of communication opportunity, the TDG allowed dictators and recipients to communicate via an online dialog box, whereas the IDG lacked this feature. Prior research suggests that communication may foster reciprocal expectations or emotional bonds (Sommerfeld et al., 2007), potentially diminishing the independent role of surveillance cues. Additionally, the payoff structure of the TDG was a zero-sum game, with a fixed total of tokens divided between the dictator and recipient, while the IDG allowed participants to voluntarily sacrifice personal gains for public goods, creating a non-zero-sum context. Non-zero-sum contexts may amplify norm-driven motivations (Rand et al., 2012), whereas zero-sum conflicts could suppress normative compliance. Furthermore, the decision framing in the IDG explicitly linked donations to socially valued outcomes, such as charitable causes support, whereas the TDG lacked such contextual framing. This framing effect (Dreber et al., 2013) might enhance the ability of eye cues to promote norm-consistent behavior. Thus, the current findings likely reflect interactions among multiple factors rather than recipient identity alone. Future studies should systematically control these variables (e.g., fixing payoff structures or communication rules) to clarify boundary conditions of the watching eyes effect. Moreover, the examination of social behavior is often influenced by the specific experimental paradigms employed, which may lead to inconsistent outcomes across different settings. Previous research has demonstrated that social framing effects are significant in the standard ultimatum game, but not necessarily in the dictator game (Dreber et al., 2013). Consequently, the watching eyes effect, a crucial area of research in altruistic behavior, may manifest differently within the dictator game paradigm compared to other experimental frameworks. Future research could benefit from comparative analyses of different social interaction scenarios to explore the generalizability and specificity of the watching eyes effect in various contexts.

Secondly, while both Self-Report Method and Spotlight Effect Method were employed to gauge a sense of being seen, significant differences were identified only through the Self-Report Method. These methods may not fully capture the complexities of a sense of being seen and its influence on behavior. To gain a deeper understanding of how this sense affects altruistic behaviors, future studies should innovate and compare various measurement approaches, develop more sensitive scales, or employ non-self-report measures such as behavioral indicators or physiological responses. Additionally, integrating neuroscience techniques, such as brain imaging, could offer insights into the neural mechanisms underlying the watching eyes effect on behavior and decision-making, thereby elucidating the psychological and biological processes at play (Lei et al., 2024).

Thirdly, while we inferred that normative mechanisms might be the core driving force behind the imagined eyes effect based on the experimental results, this study did not directly measure participants' cognitive changes regarding social norms. Future research could more directly test the hypothesis of norm activation by combining the Implicit Association Test or norm belief scales. Moreover, existing theories suggest that individuals are more likely to exhibit helping behavior in emergency situations (Shi et al., 2020) and that fast decision-making may rely more on implicit norms (Rand et al., 2012). If the altruistic behavior observed under the imagined eyes cue condition in this study is accompanied by significantly shorter decision response times, it could provide indirect support for this theory. However, since the experimental design did not record decision time data, we are unable to infer the nature of the cognitive mechanisms based on temporal characteristics. Future research could further explore the cognitive mechanisms of the imagined eyes effect through time pressure paradigms or reaction time analysis, investigating whether imagined eyes trigger altruistic behavior through implicit norm priming rather than explicit reputation calculation.

Finally, although the experiment balanced potential individual differences through random assignment, this study did not systematically measure participants' individual differences. Previous research has shown that generosity can vary due to individual differences in prosocial preferences (e.g., social value orientation) (Hilbig and Zettler, 2009) or reputation concern (Engelmann and Rapp, 2018). The lack of control over these variables may affect the interpretation of the results. Future research needs to include such measurements to clarify the interaction between the imagined eyes effect and individual traits.

## 5 Conclusion

In conclusion, this study demonstrates that in the IDG, imagined eye cue significantly increased individuals' donation intentions toward charities, whereas this effect was absent in the TDG. Specifically, when the recipient was a charity, participants in the imagined eye cue exhibited significantly more altruistic behavior compared to those in the imagined flower cue and no cue; however, no statistically significant differences were observed in the TDG (with anonymous strangers as recipients). These results highlight that the manifestation of the watching eyes effect depends heavily on the “social visibility” of the behavioral context–imagined surveillance cues effectively trigger norm-driven altruism only when individual actions are linked to socially evaluable outcomes (e.g., charitable donations). Although participants reported a stronger sense of being seen when exposed to the imagined eye cue than to other types of cues, this sense did not serve as a mediating factor between the imagined eye cue and altruistic behavior. Instead, the influence of the imagined eyes appeared to be more closely tied to individuals' intrinsic social norms, which motivated their pro-social actions.

## Data Availability

The original contributions presented in the study are included in the article/Supplementary material, further inquiries can be directed to the corresponding author.

## Footnotes

^1^Data analysis for both the TDG and IDG was restricted to participants who were assigned the role of dictator in the TDG.
