# Supplementary figures and images for: Imagined eye cue increased altruistic behavior toward charity instead of stranger
Source: Front Psychol. 2025 Mar 11;16:1503766. doi: 10.3389/fpsyg.2025.1503766 (PMC11933131; doi:10.3389/fpsyg.2025.1503766)

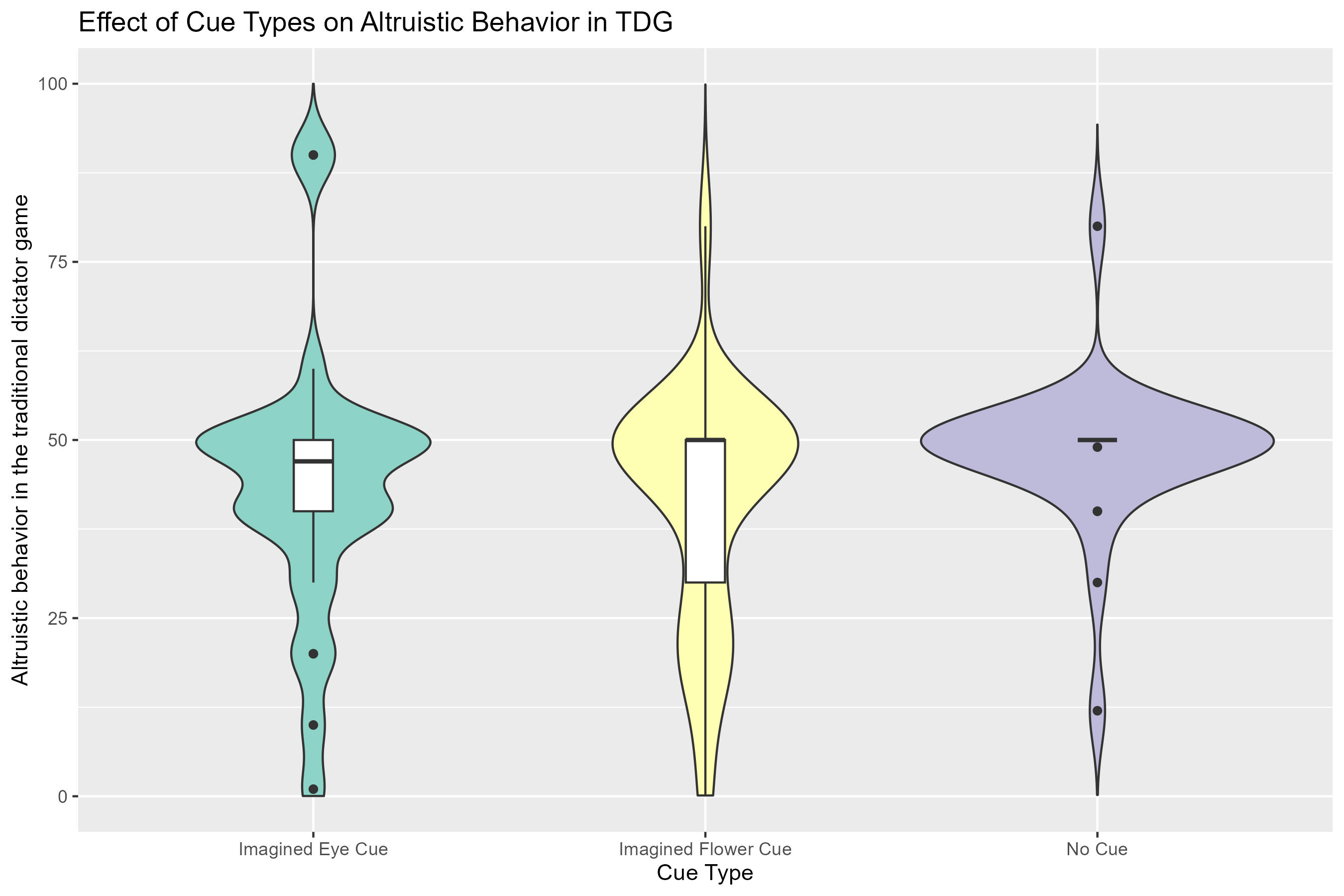

Supplement: Supplementary file 1 [file Data_Sheet_1.zip › Imagined2dataAnalysis/images/violinplot.jpg]

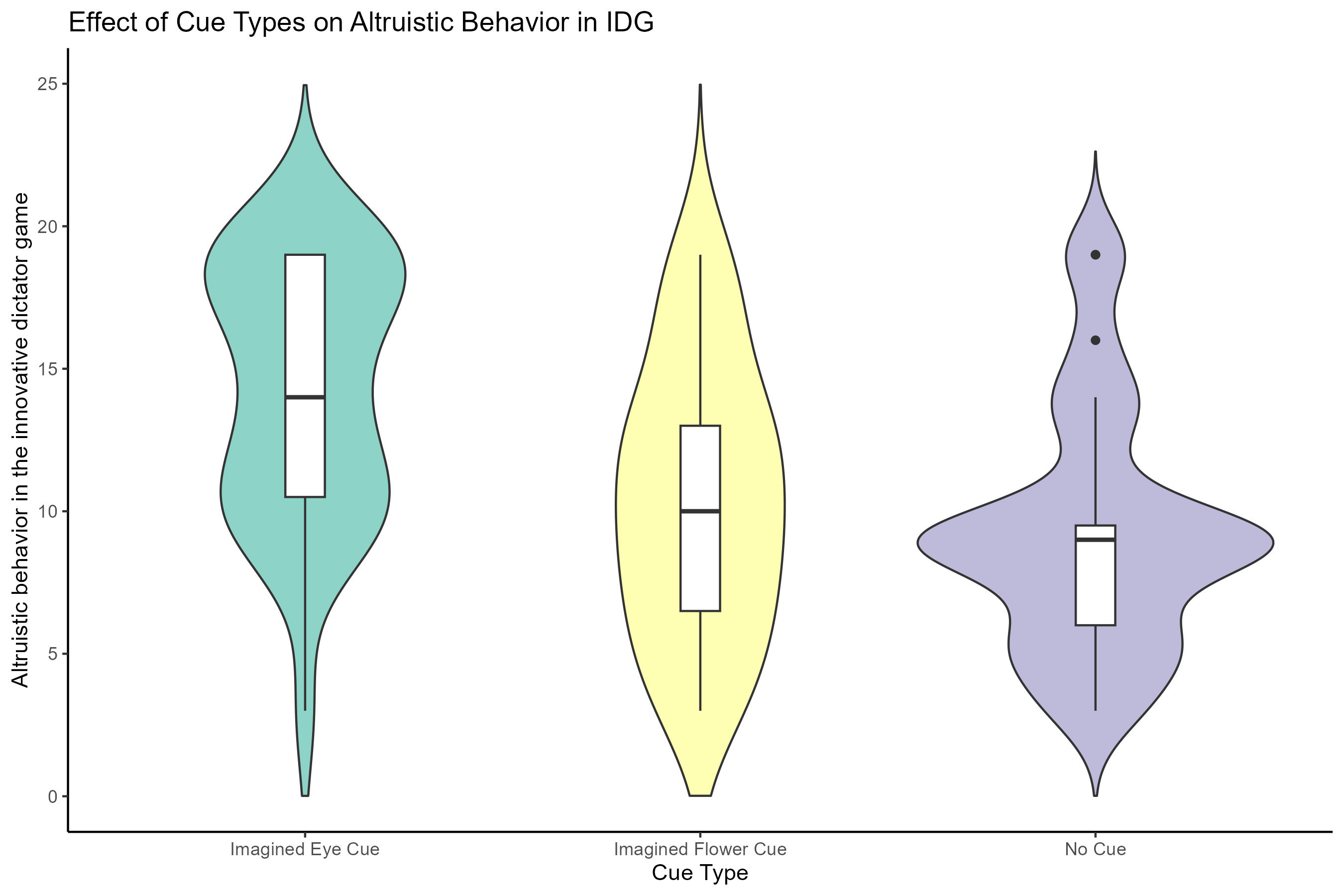

Supplement: Supplementary file 1 [file Data_Sheet_1.zip › Imagined2dataAnalysis/images/violinplotIDG.jpg]

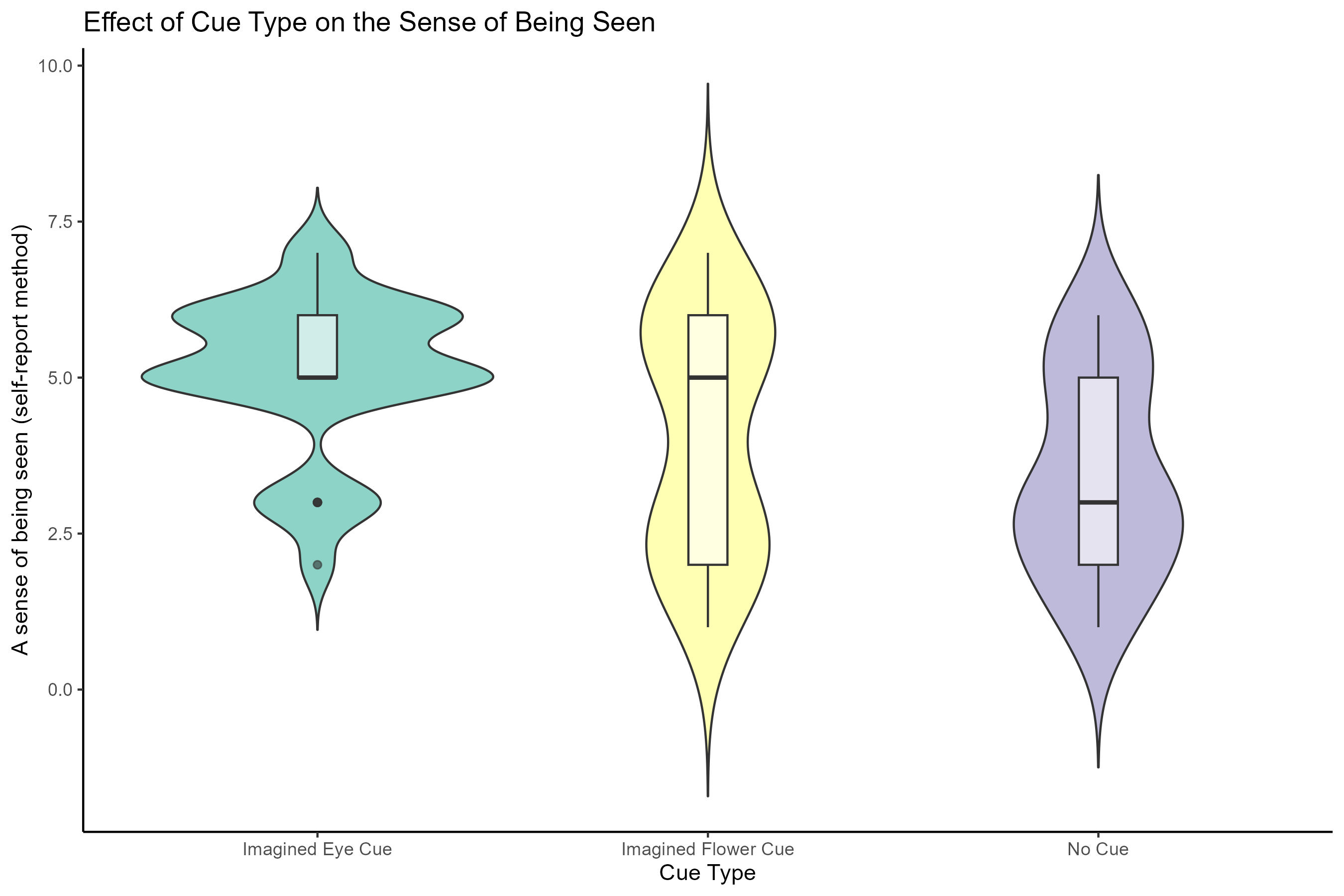

Supplement: Supplementary file 1 [file Data_Sheet_1.zip › Imagined2dataAnalysis/images/violinplotSenseSelf.jpg]

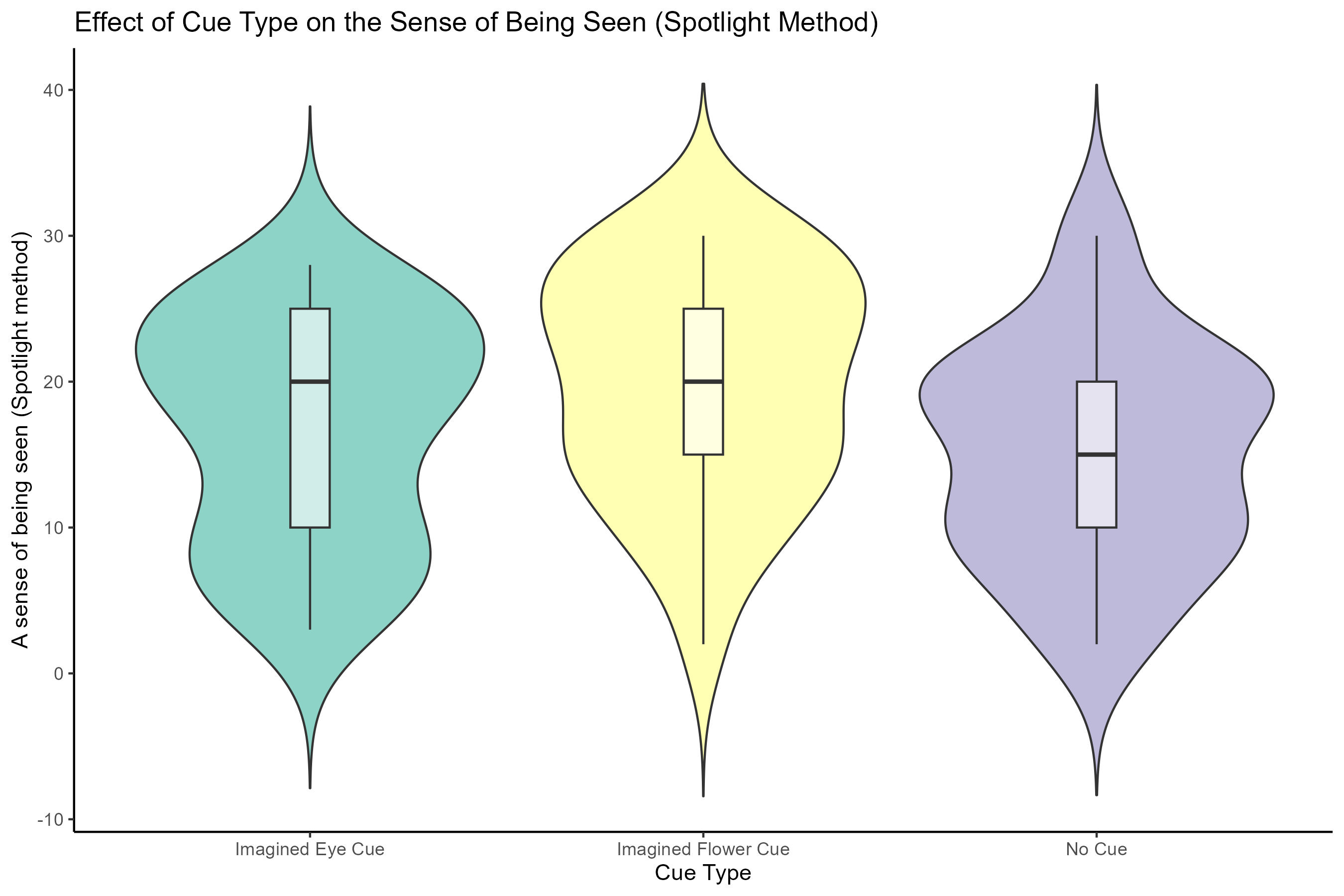

Supplement: Supplementary file 1 [file Data_Sheet_1.zip › Imagined2dataAnalysis/images/violinplotSenseSportlight.jpg]

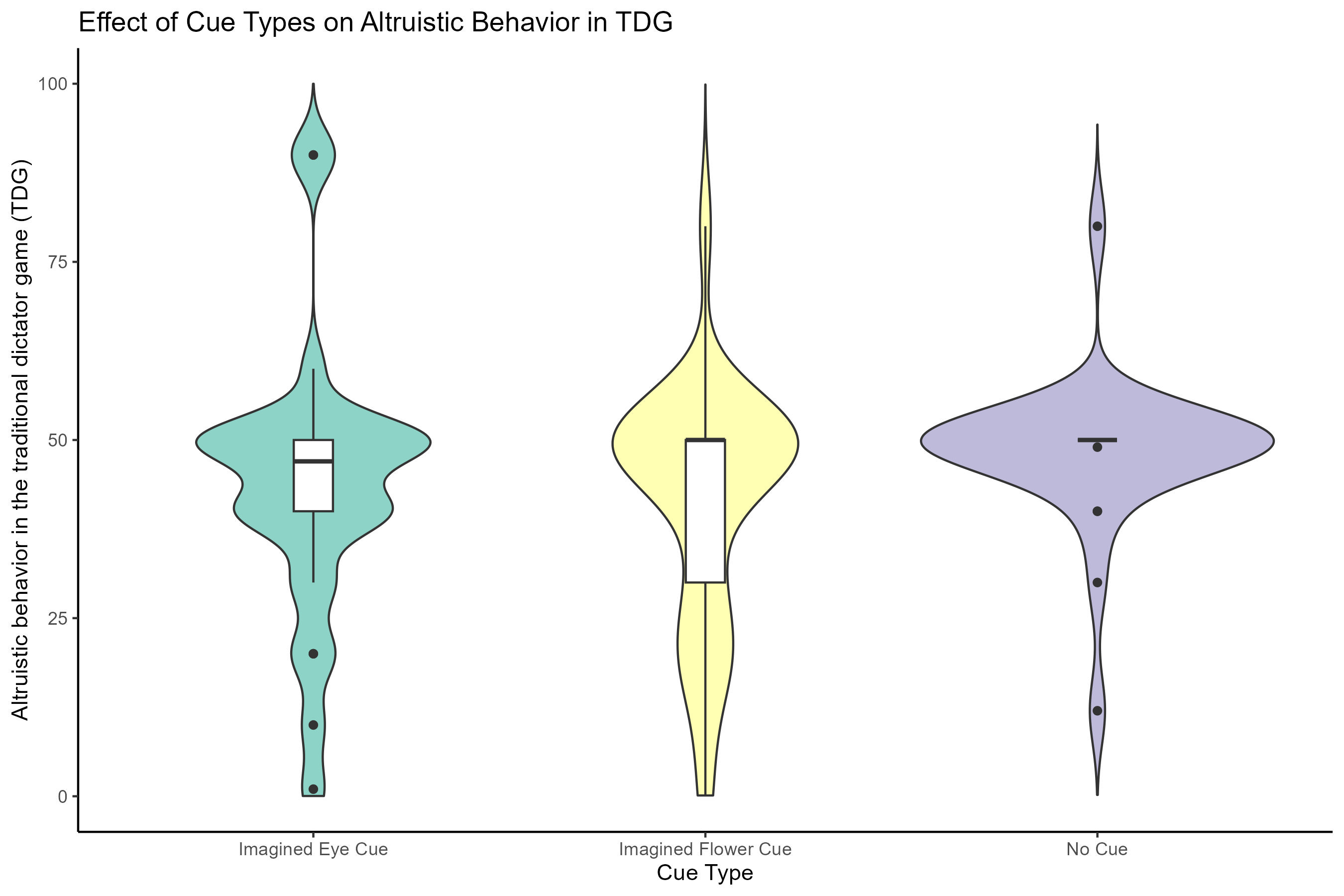

Supplement: Supplementary file 1 [file Data_Sheet_1.zip › Imagined2dataAnalysis/images/violinplotTDG.jpg]
